# Tracing human mobility in central Europe during the Upper Paleolithic using sub-seasonally resolved Sr isotope records in ornaments

**DOI:** 10.1038/s41598-020-67017-2

**Published:** 2020-06-25

**Authors:** Nina Kowalik, Robert Anczkiewicz, Jarosław Wilczyński, Piotr Wojtal, Wolfgang Müller, Luca Bondioli, Alessia Nava, Mihály Gasparik

**Affiliations:** 1 Institute of Geological Sciences, Polish Academy of Sciences, Research Centre in Kraków, Senacka 1, 31-002 Kraków, Poland; 2 Institute of Systematics and Evolution of Animals, Polish Academy of Sciences, Sławkowska 17, 31-016 Kraków, Poland; 3 Institut für Geowissenschaften, Goethe-Universität Frankfurt, Altenhöferallee 1, 60438 Frankfurt am Main, Germany; 4 Servizio di Bioarcheologia, Museo delle Civiltà, Piazza Guglielmo Marconi, 14, 00144 Roma, RM Italy; 5 DANTE Laboratory for the study of Diet and Ancient Technology, Sapienza Università di Roma, Piazzale Aldo Moro 5, Roma, 00185 Italy; 6 Department of Palaeontology and Geology, Hungarian Natural History Museum, Ludovika tér 2-6, Budapest, 1083 Hungary

**Keywords:** Geochemistry, Palaeoecology

## Abstract

Mobility of people and goods during the Upper Paleolithic has proven difficult to reconstruct given the relative rareness of remains. Nevertheless, archaeological contexts like the Late Pleistocene horizon of Borsuka Cave (Southern Poland) represent a unique opportunity to explore patterns of objects’ transportation across Central Europe. We investigated the origin of four ornaments made of European elk (*Alces alces* L.) incisors recovered at Borsuka Cave – the oldest known burial site in Poland, possibly a child grave. Laser-ablation plasma source mass spectrometric analyses of trace elements and Sr isotopic compositions revealed that one elk was roaming within a geologically uniform area while the others changed their pastures during their lifetimes. The non-local origin of the elk teeth is inferred from their exotic Sr isotopic compositions and the lack of evidence for the presence of elk in this territory during the Pleistocene. Instead, the elks’ Sr isotopic composition show good agreement with sites near the Austria-Slovakia border region and northern Hungary, ~250 km away from the study site. We argue that the artefacts were most likely brought to Borsuka Cave by humans or by a network of exchange, so far never reported in the time range 32.5–28.8 ka cal BP for Southern Poland.

## Introduction

**Figure 1:**
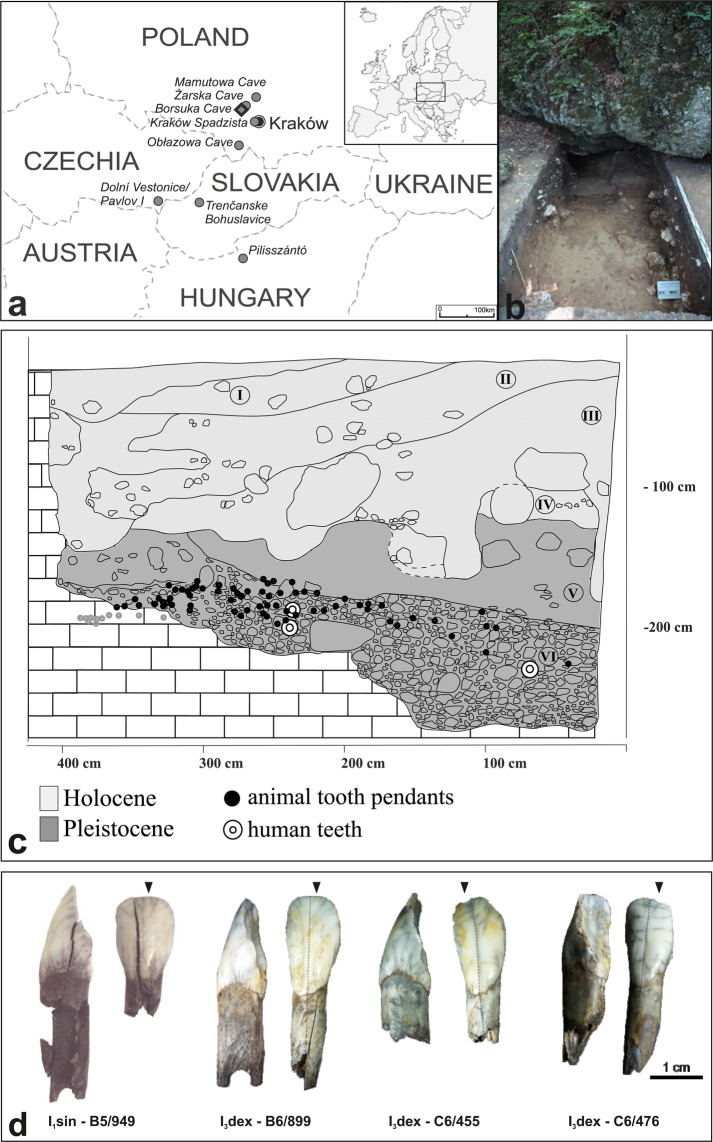
(**a**) Borsuka Cave location; (**b**) Excavation site; (**c**) Cross section of the Borsuka Cave deposits showing position of the artefacts, grey circles indicate pendants deposited in the bedrock cavity (after Wilczyński *et al*^7^.). I-IV Holocene layers, V-VII Late Pleistocene/Upper Palaeolithic layers; (**d**) European elk pendants, dashed lines mark cutting section, arrows indicate halves selected for analysis.

Burial practices involving deposition of grave goods emerged all over the European continent during the Upper Palaeolithic^1–6^. Child burials are rare^6^ and particularly valuable since they shed light on the role that children played in hunter-gatherer communities of that time^4^. Burials of individuals as young as 4 years old^1^ or even newborns^4^ associated with elaborated funerary rituals such as the use of ornaments^4^, and artefacts requiring elaborate work and time to be produced^3,6^ suggest that children were regarded as valuable members of the Palaeolithic communities^1–4^. Borsuka Cave in southern Poland potentially is one of such exceptional graves which provides a unique insight into the activity of humans north of the Western Carpathians territory during the Early and Mid-Upper Palaeolithic (35–20 ka uncal BP). Few Aurignacian and Gravettian settlements have been uncovered within this time range and the cultural affiliation of archaeological material from Borsuka Cave remains problematic^7^. Radiocarbon dates of pendants and of faunal remains from Borsuka Cave point to the Gravettian (Pavlovian) cultural period. On the other hand, the zooarchaeological record, the pendants production method and its typology have clear analogies with the Aurignacian archaeological record between 34 and 29 ka uncal BP in Lower Austria and Moravia ^8–11^. The Aurignacian culture in southern Poland, dated at 34–30 ka uncal BP is documented in Obłazowa and Mamutowa Caves (Fig. 1a), where diagnostic lithic tools along with human bone fragments were found^8,12–14^. Thus, Borsuka Cave assemblage may represent the most recent vestige of the Aurignacian tradition north of the Western Carpathians where it lasted several thousand years longer than in the Carpathian (Pannonian) Basin. Indeed, in the latter region, permanent settlements of Gravettian culture already developed in Pavlov and Dolní Věstonice^15^ (Fig. 1a).

From a geochemical perspective, personal ornaments made of animal/human teeth arguably play a special role among burial offerings. Especially the dense dental enamel portion, due to the strong resistance to *post mortem* alteration and the high potential of preserving the biogenetic composition, is useful for applying geochemical and isotopic techniques to determine their provenance/origin. Indeed, variations of the strontium isotopic composition in human and animal dental enamel corresponds well with the Sr bioavailability of the regions where the individual fed during dental ontogeny^16–22^. Additionally, tooth growth is a prolonged process capable of recording temporal changes of animal or human habits and/or place of residence^23,24^.

In this study we conducted a histological examination alongside spatially-resolved Sr isotope composition and trace element analyses of dental enamel by laser ablation (multi-collector) inductively-coupled-plasma mass spectrometry (LA-(MC-)ICPMS) in order to investigate the origin of some of the pendants made of European elk *(Alces alces L.)* incisors at Borsuka Cave dated to 32.5–28.8 cal BP^7^ (Fig. 1). We demonstrate the reliable recovery of Sr isotope composition at sub-seasonal resolution from dental enamel recording temporal changes of elk’s pastures. Additionally, by determining regional variations in Sr isotope composition using teeth of *quasi* contemporaneous non-migrating rodents, we suggest that the elks’ teeth most probably originated in the southern region of the Western Carpathians and were likely brought by humans in the Borsuka Cave area, due to the known limited mobility of the elks. This evidence makes Borsuka Cave an important reference site for deciphering artefacts’ and human mobility patterns during the Upper Palaeolithic.

## Study site and artefacts

Borsuka Cave is located in the Szklarka valley in southern Poland, about 20 km north-west of Kraków, in the southern part of the Kraków-Częstochowa Upland (Fig. 1a,b). It was discovered over a decade ago^25^ in an area which has provided a wealth of archaeological and paleontological findings^7,26–36^ dating to the Late Pleistocene. During the excavations conducted between 2008 and 2010 seven layers grouped into two sequences were identified (Fig. 1c). The first sequence (layers I-IV) was dated at the Holocene and the second sequence (layers V-VII) was dated at the Late Pleistocene^26^. The most important findings come from layer VI at the depth of 150 cm, where – among about 2000 bone fragments – 112 pendants made of teeth of large herbivores and six human deciduous teeth were discovered^26^. According to Wilczyński *et al*.^29^ the human teeth belonged to a single individual aged at death between 12 and 18 months. Although the original distribution of artefacts was disturbed, it seems likely that the child’s body was placed on the ground surface along with the burial offering. Post-depositional events possibly related to carnivore activity did not allow for the preservation of the child skeleton. As mentioned above, a precise cultural classification of layer VI is not possible due to the absence of diagnostic lithic tools. The selection of animals from which the adornments were made (large ungulate) and technique of pendants production allow to only make a cautious suggestion that they might relate to Aurignacian assemblages^29,37^. Radiocarbon dating of fragments of European elk (*Alces alces*), steppe wisent/aurochs (*Bison priscus*/*Bos primigenius*) incisors and reindeer *(Rangifer tarandus)* metatarsus gave 25150 ± 160 years BP (29589–28796 cal BP), 27350 ± 450 years BP (32503–30711 cal BP) and 26430 ± 180 year BP (31028–30340 cal BP), respectively^29^ (ages calibrated using OxCal v 4.3 software^38^ and the IntCal13 calibration curve^39^). Direct dating of the infant remains was not possible due to the unavailability of the deciduous teeth for destructive analysis. Although the resolution of radiocarbon dating is insufficient to determine whether the studied elk incisors are contemporaneous, they unequivocally prove the Upper Palaeolithic age of the artefacts found in layer VI (Fig. 1c).

The focus of this study are four pendants made of European elk incisors, one left central lower incisor (B5/949) and three right third lower incisors (B6/899, C6/455, C6/476) belonging to at least three different individuals (Fig. 1d). All incisors show various degrees of secondary post-depositional modifications like discolorations, cracks or fractures which appear more frequently on the lingual aspect (Fig. 1). The analysis of dental wear patterns indicates that the teeth most likely belonged to animals younger than 3.5 years^40^.

In order to determine the local Sr isotopic signature, we analyzed the dominant rock types (limestone and loess) collected from the site, and 15 teeth of non-migrating rodents found in the same layer as the studied pendants because of their natural ‘averaging mechanism’^41^.

## Results

### Rates and duration of the incisors’ enamel formation

**Figure 2:**
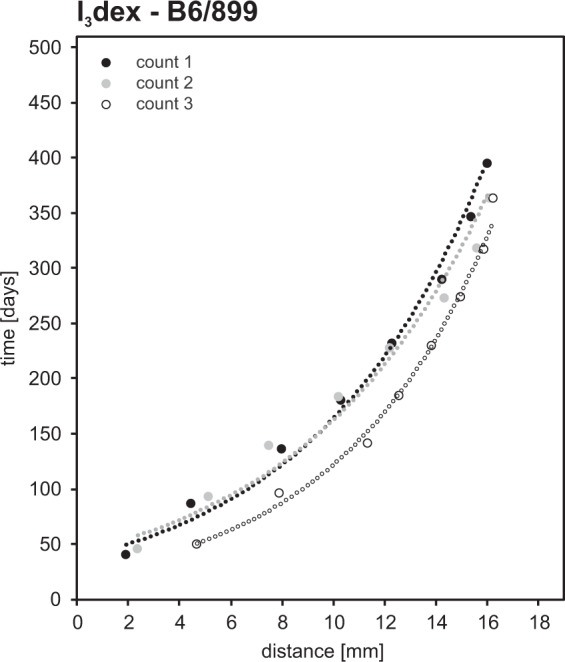
Estimated enamel daily extension rates in the incisor B6/899.

Histological investigations carried out on the incisors B6/899 estimated the daily enamel secretion rate (i.e. the speed at which the enamel forming cells secrete the matrix along the crown thickness^42^) as 5 µm day^−1^ and it appears to be almost constant along the whole length of the dental crown. The enamel extension rate (i.e. the speed at which ameloblasts are recruited along the Enamel Dentine Junction - EDJ^42^) gradually decreases towards the cervix from about 83–60 µm day^−1^ to about 12–8 µm day^−1^, which is associated with the gradual increase of the angle between the Retzius lines and the EDJ from about 2–3 degrees near the crown tip up to 25 degrees near the cervix. Three rounds of repetitive counting indicate that the Crown Formation Time (CFT) was about 360–390 days (Fig. 2). Thus, the estimated time of the whole incisor formation is greater than one year.

### Trace elements

The main goal of the LA-ICPMS elemental analyses was to identify the degree of geochemical alterations and to evaluate their potential influence on the *in-vivo* Sr isotope composition.

**Figure 3:**
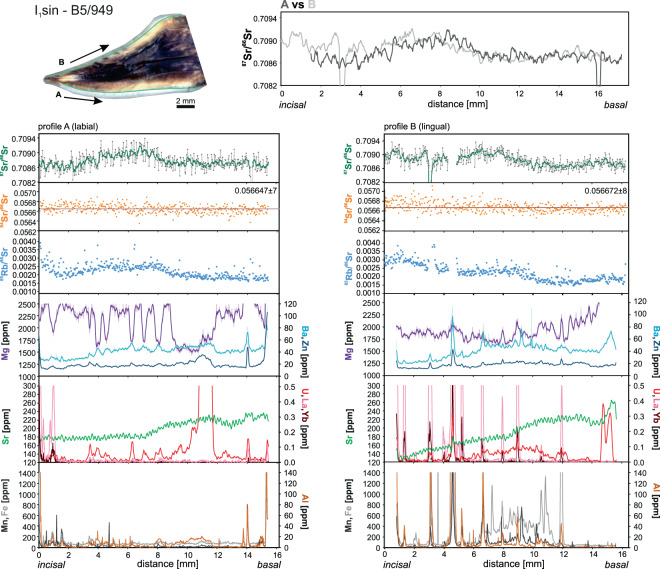
Strontium isotope composition and selected minor and trace element concentrations extracted from the incisors B5/949. Diagrams on the left refer to the labial side while diagrams on the right refer to the lingual side of the incisors. Trace element and Sr isotope composition measurements conducted along the same lines marked in the photographs. The top graphs show superposed smoothed ^87^Sr/^86^Sr measurements (moving average method with a span of 5) in both sides of the incisors.

**Figure 4:**
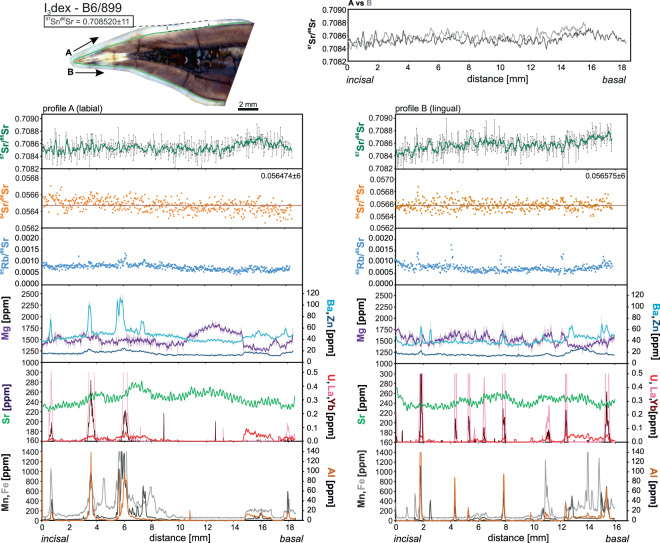
Strontium isotope composition and selected minor and trace element abundances extracted from the incisor B6/899. For explanation see caption to Fig. 3.

**Figure 5:**
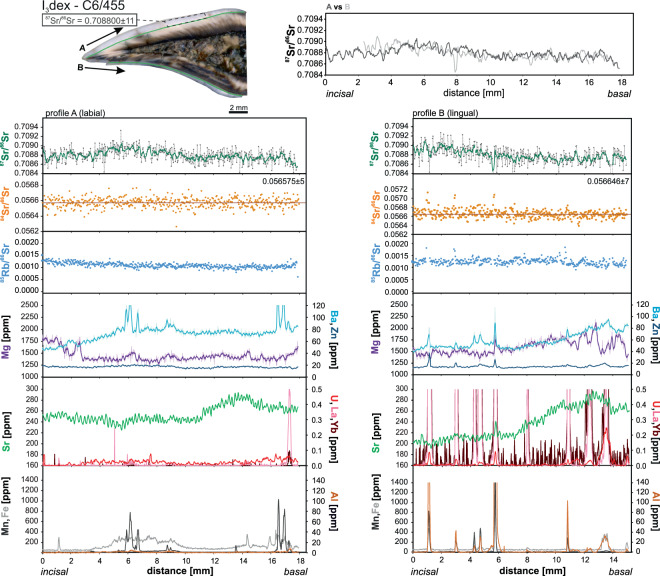
Strontium isotope composition and selected minor and trace element abundances extracted from the incisor C6/455. For explanation see caption to Fig. 3.

**Figure 6:**
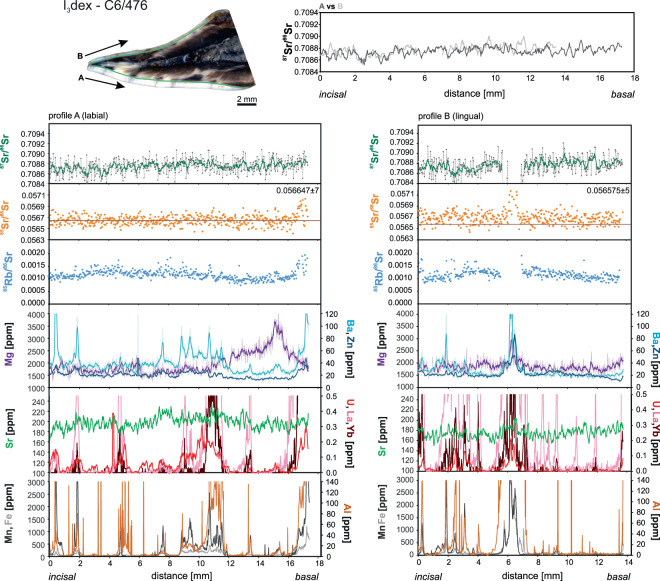
Strontium isotope composition and selected minor and trace element abundances extracted from the incisor C6/476. For explanation see caption to Fig. 3.

Nearly all the analyses show significant variations in the concentrations of minor and trace elements along the dental enamel when moving from the incisal border towards the cervix. Additionally, there are some differences between the labial and the lingual side of the same tooth (Figs. 3–6). The largest differences we observed in the incisor B5/949, where Mg in labial side (profile A) shows numerous peaks and troughs inversely correlating with abundance of Ba and U and, to a smaller extent, Zn. These patterns do not match for all its length with those on the lingual side (profile B) where variations in Mg are much smoother and dominated by a clear rise near the basal part. There is no obvious correlation between the Mg content and any other measured element on the lingual side (Fig. 3). Manganese, Fe and Al locally show peaks or spikes on both sides of the incisor that correlate with the stained or fractured regions. They typically also correlate with rare earth elements (REE), rarer U concentration. In profile B there is a prominent hump in the middle of the section where U, Fe, Mn and Al show higher concentrations. Zinc, Ba and Sr show very similar abundance and trends on both sides of the incisor with a notably lower Sr content near the incisal line A. The three remaining incisors qualitatively display similar features (Figs. 4–6). The major difference is that they show much less variation between the labial and lingual sides. Spikes of Mg, Ba and Zn typically correlate with U, REE and/or Mn, Fe and Al, as well illustrated by the incisor C6/476 (Fig. 6). The same pattern was recorded on both sides of the tooth. The differences in the abundance of Sr in the incisal and basal sides are rather small and do not correlate with the changes observed among other elements (Fig. 6).

### Isotopic composition of Sr in elk incisors

*In situ* LA-MC-ICPMS Sr isotope analyses are summarized in Figs. 3–6 and Supplementary Table S3. Strontium isotope composition profiles extracted from the specimen B5/949 in both labial and lingual sides show smooth variations in ^87^Sr/^86^Sr ratios from the incisal to the basal margins (Fig. 3). The range of ^87^Sr/^86^Sr values is fairly large and varies from 0.7087 to 0.7093 (Fig. 3). For incisor B6/899, the Sr isotopic composition is more homogenous, nearly constant throughout most of the ablation path, except for the basal region where higher values are recorded (Fig. 4). The corresponding ^87^Sr/^86^Sr ratios are generally lower (0.7085–0.7088) and just overlap with the lowest values observed in the described above incisor B5/949 (Fig. 4). The incisor C6/455 recorded a Sr isotope composition entirely within the range observed in B5/949 but the fluctuations are smoother, resembling those in B6/899, with a range of ^87^Sr/^86^Sr ratios between 0.7087 and 0.7091 (Fig. 5). Finally, incisor C6/476 shows homogenous pattern with ^87^Sr/^86^Sr values ranging from 0.7086 to 0.7091 (Fig. 6). This compositional range well overlaps with the values recorded in the incisors B5/949 or C6/455 (Figs. 3 and 5).

In general, the ^87^Sr/^86^Sr ratios recorded in both labial and lingual sides are in remarkable agreement for each incisor. There is, however, one exception in the incisor B5/949 where there the two patterns in the first 3 mm near the incisal margin do not correlate well.

In all samples, the naturally invariant ^84^Sr/^86^Sr ratio appeared stable over the entire length of the ablation track (Figs. 3–6) and yielded accurate within uncertainty value of 0.05660 ± 13 (error is 2 standard deviations and relates to the last significant digits). This value is particularly sensitive to Kr interferences and its accuracy reassures the correctness of the applied methodology^24^. The most significant correction, however, is necessary for the ^87^Sr isotope whose measurement is interfered by ^87^Rb, given the relatively elevated ^85^Rb/^86^Sr ratios on the order of 0.5–4 ×10^−3^ in our samples. Accuracy of the performed correction was verified by microsampling (drilling) of enamel from two randomly chosen incisors followed by solution-based analysis. The results are presented in Supplementary Table S2 and on Figs. 4 and 5 where we marked sampled areas. Conventional and *in situ* analyses agree well which confirms the accuracy of the correction procedure described in Müller and Anczkiewicz^24^ and reassures accuracy of our *in situ* analyses.

### Regional Sr isotopic signature

**Figure 7:**
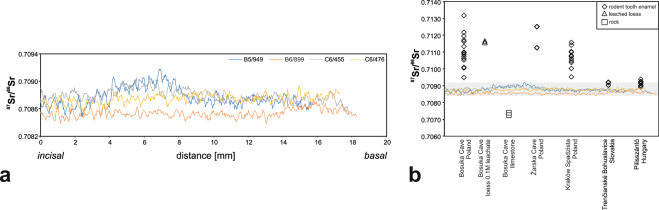
(**a**) Comparison of smoothed, incisal to basal ^87^Sr/^86^Sr paths determined by LA MC ICPMS from the labial profiles of all the studied incisors; (**b**) Comparison of Sr isotope composition of the elks’ incisors with rodents from Borsuka Cave, Żarska Cave, Kraków Spadzista site in Poland, Pilisszántó site in Hungary, Trenčianské Bohuslavice site in Slovakia and with the dominant rock types in Borsuka Cave (limestone and loess). See text for details.

The main Sr providers are local rocks which are composed predominantly of limestone and, to a lesser extent, loess deposits. Limestone is mineralogically simple and its whole rock analysis well reflect Sr composition released to soil and water. Expectedly, Jurassic limestone analyses yielded low ^87^Sr/^86^Sr ratios ranging from 0.7072 to 0.7074. Average bulk rock analyses of loess, on the other hand, yielded a highly radiogenic ^87^Sr/^86^Sr ratio of 0.7330. However, loess is a more complex rock and its minerals (mixture of silicates with lesser amount of carbonates and phosphates) show variable degree of resistance to chemical breakdown. Highly radiogenic Sr in loess is linked to relatively insoluble silicates. In order to check the isotopic composition of Sr of a more soluble loess fraction composed of carbonates and phosphates more readily available to plants, we conducted 0.1 M acetic acid leaching. As a result, we obtained ^87^Sr/^86^Sr ratio of 0.7116, considerably lower than the bulk rock composition (Fig. 7b and Supplementary Table S2).

A different practice of determining local biological Sr signature is to analyze tissues of non-migrating rodents from the same archaeological strata^41^. Analyses of the Pleistocene rodents from the pendant-bearing layer VI of the Borsuka Cave (Fig. 1) showed a rather wide range of ^87^Sr/^86^Sr ratios between 0.7095 to 0.7132 (Supplementary Table S2 and Fig. 7b). Additionally, we investigated the Sr isotope composition of rodents from the two Upper Palaeolithic sites on the southern side of the Carpathians in Trenčianské Bohuslavice (Slovakia) and Pilisszántó Cave (Hungary), the proximate sites where elk was already known during the Last Glacial Maximum (LGM). In both sites ^87^Sr/^86^Sr is less radiogenic, ranging between 0.7090–0.7092 in Slovakia and 0.7089 and 0.7094 in Hungary.

## Discussion

### Enamel formation rates

The studies on dental enamel formation mode and time have been often focused on hominids^43–45^. However, histological studies of other mammals have become more frequent and similar studies for Equidae, Proboscidae, Rhinocerotidae or Cervidae are now available^46–52^. The results of our histological analysis of the incisor B6/899 agree well with observations made on other mammals. The mean daily secretion rate is estimated at 5 µm day^−1^ which is in the broad range of published estimates for horse teeth, which vary from 5^46^ to 18^52^ µm day^−1^. More closely related to elk, sika deer^47^ shows much larger mean daily growth of 10.6 µm day^−1^, which supports the inference of Dirks *et al*.^50^ who pointed out that this parameter is primarily controlled by tooth size, morphology, phylogenetic position and individual life history. Similarly to the previous studies^49,50,53^ our estimates indicate the gradual decrease of enamel extension rates towards the tooth cervix. By counting circadian and circaseptan growth increments, we determined the approximate CFT as greater than one year (Fig. 2) which is sufficiently long to make incisor teeth valuable providers of information on temporal changes of pastures, and mobility patterns.

### Trace elements

It has long been recognized that while dentine and cementum are prone to secondary alterations, fossil dental enamel is often capable of retaining *in vivo* chemical and isotopic composition^54–60^. Our analyses show large fluctuations in the abundance of Mg, Ba and, to a lesser extent, Zn. Uptake of these elements is controlled physiologically or by diet, and thus the fluctuations are likely to reflect the original biological record rather than *post mortem* processes. This inference is supported by the general lack of correlation of the observed changes in Mg, Ba and Zn with elements considered to be markers of the secondary alterations like Mn, Fe, Al, U or REE (Fig. 3). There are some zones, however, where such correlations are obvious and document post-enamel formation alteration. On labial side of the incisor B5/949, deep Mg troughs correlate with the slightly elevated U content (Fig. 3). Although trace amounts of U can easily be incorporated into apatite via food and water, U peaks also correlate with higher Al concentration. In the same tooth on the lingual side, a broad U-hump correlates with higher Al, Fe, Mn and Ba as well as with the opposite trend in Mg (Fig. 3). The nature of the described correlations in the labial and lingual sides seems to be different, though. While changes in profile A are confined almost exclusively to very narrow zones and most likely reflect pollution by sediment within the micro-cracks (as indicated by elevated Al content), in profile B the altered zones are much broader and correlate with decolorized areas where changes are likely to have resulted from diffusional exchange facilitated by micro damages whose presence is chemically expressed by numerous spikes in Mn, Fe, Al and U within the hump region^58,59,61–67^. These two types of processes leading to the observed geochemical patterns are also present in the other incisors, which overall show a better degree of preservation (Figs. 4–6). Alterations are more common on the lingual sides and in the cervix regions where enamel layer is thinner and weaker (Figs. 3–6). In the preserved parts of enamel Al, Fe, Mn and REE abundances are near or below the detection limits (sub-ppm level) and Sr, Ba and Zn show fairly stable concentrations. However, even in “clean” enamel, we still observe small but very clear differences in the chemical record between the labial and lingual sides of the same incisor (Figs. 3–6). This could be explained by slightly different mineralization time between the two sides.

Interestingly, and rather surprisingly, even in the regions of quite severe alterations, Sr abundance does not seem to be affected. The only potential exception is confined to strongly fractured regions like that in profile B in specimen C6/476 (Fig. 6). However, even there the changes are subtle only. This may suggest that interaction with soil and/or fluids circulating in the burial place had no significant influence on the Sr record. Strontium content in such fluids is considerably lower than abundance of Sr in the enamel^68^ and thus, only its significant incorporation or loss would be detectable. Additionally, if fluids circulating at the burial site were responsible for changes in the abundance of Sr in dental enamel, its isotopic composition should be shifted towards the local values determined by the rodent analyses. However, the local isotopic composition of Sr is significantly different from that in the original and altered enamel and incorporation of significant amounts of Sr of different isotopic composition would easily be detectable. Therefore, if any incorporation of local Sr took place, it is negligible.

Thus, accepting that the observed Sr abundances reflect original, *in vivo* records in dental enamel, changes in Sr observed in specimens B5/949 and C4/455, must reflect changes in pastures probably due to temporal variations in availability of food. This implies that the animal moved to a different location with different geochemical signature and/or fed on different vegetation (see also Kohn *et al*.^69^). This is in accord with the observations made on modern European elk which consume berries and green foliage in summer, and in winter, when grasslands are covered with snow, their diet is composed of leaf litter and highly lignified wood stems^70,71^.

### Sr isotopic composition

Despite some localized alterations described above, we observe an excellent match of the ^87^Sr/^86^Sr record between the labial and lingual sides of each incisor (Figs. 3–6). This not only demonstrates the reproducibility of our analyses but also shows that they reflect pristine enamel compositions. The only disputable exception is small fragment of the incisor B5/949 where near the incisal part (first 3 mm of the ablated path) ^87^Sr/^86^Sr ratios in labial and lingual sides diverge (Fig. 3). In view of a fact that alteration indicating elements (Al, Fe, Mn, REE) do not exhibit variation corresponding with the “too high” ^87^Sr/^86^Sr ratio, we find *post mortem* mixing with the local Sr unlikely. The most probable reason for the observed discrepancy is the particularly thin enamel layer in this part of the incisor, which with the resultant imprecise positioning of the ablation path, locally involved mixed ablation of enamel with small volumes of dentine whose composition is indeed more radiogenic (0.7099–0.7105).

After superimposing the smoothed ^87^Sr/^86^Sr profiles of all four incisors measured on the labial sides, incisors B5/949 and C6/476 show nearly identical patterns (Fig. 7a).The resemblance of those two records suggests that the two incisors may even come from the same individual. Taking into account over a 1 year duration of the enamel formation, the fluctuations in ^87^Sr/^86^Sr ratios recorded in C6/455 and B5/949 certainly reflect temporal (about 6 months based on CFT) changes of the pasture areas. The incisor C6/476 shows nearly constant Sr composition, which we inferred to indicate a more sedentary behaviour or limited mobility within an area of invariant Sr isotopic composition. Nevertheless, the rather high similarity in Sr isotope composition of the three incisors strongly suggests that elks originated from the same region. The isotope composition of Sr in the incisor B6/899 is somewhat lower (Fig. 7a) but taking into account the partial overlap with the three other incisors, this individual probably spent most of its time in the same region, which only was a seasonal destination for the two (or three) other individuals described above. Little difference in Sr isotope composition implies that all four incisors belonged to elks hunted in a geologically rather uniform area, distinct from that of Borsuka Cave. The proposed interpretation is in line with the observations conducted on modern elk which indicate that some of them are migratory and some are local. They may also change their habitat seasonally or stop migrating for a few years^72–74^.

### Allogenous origin of the artefacts

Mammalian herbivores assimilate Sr into their bones and teeth primarily via plants which contain considerably more Sr than water. Hence, biologically available Sr is linked to soil formation processes and to its assimilation by vegetation. Different approaches have been applied towards establishing regional biogenic Sr background. Analyses of rocks, plants, bones, dental enamel, soils or water have been used^75–86^. While bones are very susceptible to diagenesis, plants, soils and water are likely to be influenced by anthropogenic activity (e.g. farming). Additionally, post-glacial sedimentary overprint in the study area (besides possible industrial contamination), makes recent plants of little use for determining local isotopic signature around Borsuka Cave ~30 ka ago. Thus, combined analyses of local geology with analyses of the highly resistant dental enamel of archaeologically-contemporaneous local rodents, despite some differences in diet and range in comparison to large herbivores, we consider as the best available approach for determining the local Sr isotopic composition around Borsuka Cave.

At Borsuka Cave, the mixture of Sr isotopes derived from dissolution of limestone and the more soluble fraction of loess almost certainly dominated the local biologically-available isotopic composition of Sr. However, due to complex soil formation processes and unknown contribution of each source, on the basis of the geological context alone, we cannot draw any conclusions about the exact ^87^Sr/^86^Sr ratio that was assimilated by the local animals. Thus, analyses of rodents derived from the same sediment strata as elks’ remains, despite some limitations mentioned above, are a more reliable approximation of the isotopic composition of locally bioavailable Sr.

**Figure 8:**
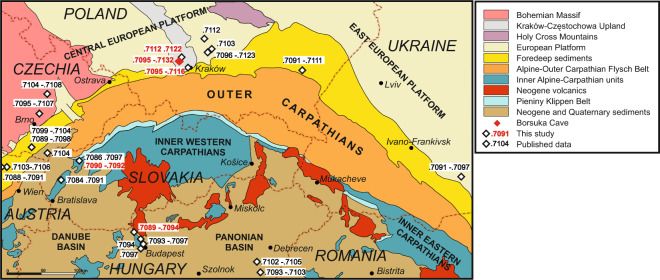
Regional Sr isotopic composition variations. Sources of ^87^Sr/^86^Sr values are indicated in Supplementary Table S2. Geological map compiled using published studies^102–104^.

Our analyses of rodents found in the Pleistocene layer VI of the Borsuka Cave showed a range of ^87^Sr/^86^Sr ratios between 0.7095–0.7132, only slightly narrower than that defined by the rocks (Fig. 7b) suggesting that mixing of Sr from two dominant lithologies indeed could have dominated regional signature. Another important observation is that despite very limited mobility range of rodents, they provided widely dispersed ^87^Sr/^86^Sr ratios (Fig. 7b) revealing substantial small distance variations. This adds to an overall difficulty in deciphering mobility paths of longer distance migrants. However, the key observation is that even the lowest ^87^Sr/^86^Sr ratio measured in the rodent teeth is still significantly more radiogenic (by at least 0.0002) than the ^87^Sr/^86^Sr observed in the enamel of the studied elks’ incisors. Furthermore, the low ^87^Sr/^86^Sr value is determined just by a single analysis and the most common ^87^Sr/^86^Sr composition measured in rodents ranges from 0.7100 to 0.7120, which is considerably different from Sr composition recorded in the incisors (Fig. 7b). Acknowledging the overall paucity of biogenic Sr isotopic composition data produced so far, such low values do not seem common in Central Europe as shown in compilation of ^87^Sr/^86^Sr ratios obtained with different methods in this and the previous studies^75–81,83,84,86,87^ (Fig. 8). In the direct neighbourhood of Borsuka Cave, some ten to several tens of km, all known Sr isotopic compositions are similar to those of our study, and thus, are significantly more radiogenic than isotopic composition of the measured elks’ incisors^84^ (Fig. 8). Only four areas located far south or southwest of the studied site show Sr isotopic composition overlapping with that found in the elks’ incisors: Trenčianské Bohuslavice and Tmavá skala cave in Slovakia, Pilisszántó in Hungary and Henzig in Austria^80,81,86^(Fig. 8). Our results indicate that the pendants did not originate from locally hunted elks and that the artefacts could be indicators of exchange or human regional mobility. This is in accord with the paleogeographic and paleozoological studies which, so far, have not provided any evidence for the presence of elk in this region at 30–27 ka BP^88^, despite suitable flora and climatic conditions^89^. However, the fossil record shows that elk was already present at that time in neighbouring regions of the Czech Republic, Hungary, or, more distant, Moldavia, North-Western Balkans, and Northern Italy^90^.Thus, it is probable that the Upper Palaeolithic elk whose teeth have been found in Southern Poland roamed in areas similar in geology and ecology to southern regions of the Western Carpathians and that their teeth were brought to the Borsuka Cave as hunters’ trophies or were exchanged between human groups.

## Conclusions

We conducted detailed histological, trace element and Sr isotope composition analyses aiming at determining the origin of the four pendants made of elks incisors excavated in Borsuka Cave in southern Poland, the earliest burial site on Polish territory, dated at 32.5–28.8 ka cal BP^7^. Our histological study estimated the duration of a single incisor formation as greater than one year. Such time of the enamel formation allowed to achieve sub-seasonal record of trace element and isotopic measurements in dental enamel using high spatial-resolution laser-ablation mass spectrometry. Laser ablation ICPMS trace element analyses revealed two types of chemical alteration of pristine dental enamel, which are associated with 1) localized mechanical damage zones subsequently filled by sediment/soil particles, and 2) broader areas modified by possible diffusional/adsorption changes, locally enhanced by mechanical damage. Despite the observed changes, nearly all enamel preserved biogenic Sr isotopic compositions which document temporal changes of pastures by the studied elks or, in the case of one sample, more stationary behaviour. All the studied incisors show the same or very similar ^87^Sr/^86^Sr ratios of 0.7085–0.7093 indicating the same or proximate feeding areas. The Sr isotope composition of the Pleistocene non-migrating rodents from Borsuka Cave unequivocally excludes a local origin of elk, which is in line with zooarchaeological evidence. Conversely, our analyses show good agreement with sites in Slovakia and Hungary. This indicates that the artefacts were likely brought to Borsuka Cave by humans coming from the southern side of the Western Carpathians or by a network of exchange, so far never attested in the time range 32.5–28.8 ka cal BP in Southern Poland.

## Materials and methods

### Enamel growth rate estimates in elks’ incisor

Dental enamel formation is a two stage process consisting of matrix secretion followed by a maturation one when most of the mineralization occurs^60^. Enamel growth is appositional and is characterized microscopically by circadian rhythm markers (cross striations) and by longer period markers^42^ (Retzius lines). In order to use these features for determining rates and extent in time of the dental enamel formation processes, we first prepared petrographic thin sections which enabled good visibility of the above mentioned growth features under optical microscopy (Axioscope Zeiss equipped with a digital camera). We took a series of high resolution microphotographs of the dental enamel in the best preserved incisor B6/899 which was subsequently analyzed using ImageJ2 software^91,92^. The measurements were conducted on the labial side where enamel is ~50% thicker than on the lingual side and the expression of incremental growth markers much clearer. Additionally, the labial side is much better preserved in our sample. The Retzius lines are well visible near the incisal and the central part of the incisor, but become blurred or even disappear towards the cervix, making the estimates in that region more uncertain or even impossible. The crown formation time of the incisor was calculated using the method described by Dean^93^, with daily secretion rates directly measured for each prism segment. The crown formation time of the tooth was estimated independently 3 times.

### Preparation of artefacts for *in situ* analyses

The incisors B6/899, C6/455 and C6/476 were sectioned across their central part, in the labio-lingual direction. The fourth incisor B5/949 was split into two pieces along a naturally developed fracture (Fig. 1d). Subsequently, the incisors half-pieces were mounted in an epoxy resin and polished with a diamond paste. The prepared mounts were used for spatially-resolved trace element and Sr isotope composition measurements.

### *In situ* trace element and Sr isotope composition analyses

All analyses were conducted at the Geochronology and Isotope Geochemistry Laboratory of the Kraków Research Centre, Institute of Geological Sciences, Polish Academy of Sciences. Spatially-resolved analyses were performed using a RESOlution M-50 excimer (ArF 193 nm) laser ablation system (formerly Resonetics, now Applied Spectra) equipped with a dual volume S155 sample cell coupled to the following different mass spectrometers.

For trace element analyses, the LA system was coupled with an ICPMS XSeriesII (ThermoFisher). The analyses were performed in line-scan mode nearest to the enamel-dentine junction (EDJ), starting from older, incisal surface and proceeding towards the younger root (Figs. 3–6). The EDJ side was selected because full mineralization of the inner and the middle enamel layers are completed shortly after the matrix formation while the complete maturation of the enamel in teeth may finalize weeks to months after matrix formation^46,60^. Thus, sampling of the inner enamel side (near the EDJ) avoids much of the dampening effect which is observed in the outer enamel layer^60,94^. A laser spot of 67 µm diameter with an energy density of about 7–8 J/cm^2^ and 15 Hz frequency were used to ablate the sample; a x-y-stage speed of 2 mm/min was used (Supplementary Table S1). External standardization utilized NIST SRM 612, using values of Jochum *et al*.^95^. The stoichiometric calcium concentration of 37% m/m was used as internal standard and MPI-DING glasses^95^ were measured to assess data accuracy. Data reduction was performed using Iolite v.2.31 software^96–98^ which operates within IGOR Pro environment (WaveMetrics, Inc.).

*In situ* Sr isotopic analyses were conducted using the same LA system connected to a MC-ICPMS Neptune (ThermoFisher). Detailed description of our setup and data reduction approach can be found in Müller and Anczkiewicz^24^ where we provided test results showing the reliability and robustness of the conducted corrections on critical isobaric interferences caused by Kr and Rb isotopes, doubly charged REE^++^ ions, Ca dimers and polyatomic interferences (e.g. CaPO, CaAr). The key analytical parameters specific to this study are summarized in Supplementary Table S1 and described below. Similar to the trace element measurements, the analyses were performed in line scan mode along the same lines as trace element analyses with a laser beam size of 120 µm, 20 Hz laser repetition rate and a fluence of about 7–8 J/cm^2^. A slow x-y-stage speed of 0.5 mm/min was chosen which - at [Sr] of ~200 µg/g (see 4.2) - yielded 3 × 10^−11^ A ion currents and thus satisfactory analytical precision and spatial resolution. Data quality was monitored by frequent measurements of tooth enameloid of a modern shark whose ^87^Sr/^86^Sr = 0.709170 ± 46 (2 standard deviations of unweighted mean; n = 14) agrees well with the expected present day sea water composition^87^Sr/^86^Sr = 0.709175^99^. We measured both labial (A) and lingual (B) sides of each tooth which grow contemporaneously, and thus, are expected to provide similar Sr isotopic compositions. Such replicate measurements served as an additional data quality control. Additionally, we monitored invariant ^84^Sr/^86^Sr ratios (see below). Trace elements and Sr isotope composition profiles were smoothed using moving average method with a span of 5 (Figs. 3–6).This value reduces excessive noise of time resolved analyses but preserves all chemical or isotope composition variations at high resolution.

### Conventional Sr isotope composition measurements

Two samples prepared out of elks’ incisors aiming at verifying accuracy of *in situ* Sr isotopic composition analyses were prepared using microsampling technique. This involves drilling of dental enamel (Figs. 4 and 5) with a fine dental drill which helps to avoid contamination by dentine. So created powder were dissolved in 2 M acetic acid and subjected to two stage columns chemistry described below.

Rodents teeth preparation was more involved. Because their enamel layer is very thin, typically below 30 µm precise *in situ* Sr isotope composition measurements by LA-MC ICP-MS are problematic due to the lower amount of ions detected and consequently result an unsatisfactory precision. Precise microdrilling would also be problematic. Hence, we applied conventional wet chemical analyses of bulk tooth enamel. In order to eliminate, or at least minimize, mixing of tooth enamel with dentine we carried out manual, mechanical separation followed by chemical leaching. Mechanical separation was carried out using simple tools like fine tip tweezers and a needle. Subsequently, we applied chemical leaching according to modified procedures of Lee-Thorp *et al*.^100^ and Müller *et al*.^101^. Firstly the enamel fragments (still containing some amount of dentine) were ultrasonically cleaned in acetone, followed by ethanol and ultra-high purity water. Subsequently, samples were treated for a few hours with 0.1 M acetic acid in an ultrasonic bath. Leachate was centrifuged and the residue was further treated with 2 M acetic acid. The second leachate was subjected to two stage columns chemistry. In the first stage AG 50W-X8 resin by Bio-Rad was used while in the second stage, Sr fraction was purified on the Sr-spec resin by Eichrom. Subsequently, Sr fractions were dried down, re-dissolved in 2% HNO_3_ and measured using the MC-ICPMS *Neptune*. Desolvating nebulizer AridusII by CETAC was used as a sample introduction system. Exponential mass bias correction conducted using ^86^Sr/^88^Sr = 0.1194. Long term reproducibility of SRM987 value is 0.710250 ± 13 (2 SD, n = 60).

## Supplementary information

Supplementary table S. 1.

Supplementary table S. 2.

Supplementary table S. 3.

## Data Availability

All data generated or analysed during this study are included in this published article (and its Supplementary Information Files).
