# Supplementary material for: Tracing human mobility in central Europe during the Upper Paleolithic using sub-seasonally resolved Sr isotope records in ornaments
Source: Sci Rep. 2020 Jun 25;10:10386. doi: 10.1038/s41598-020-67017-2 (PMC7316840; doi:10.1038/s41598-020-67017-2)
Supplement: Supplementary file 1 — Supplementary table S. 1. [file 41598_2020_67017_MOESM1_ESM.docx]

**Tracing human mobility in central Europe during the Upper Paleolithic** **using sub-seasonally resolved Sr isotope records in ornaments**

Nina Kowalik, Robert Anczkiewicz, Jarosław Wilczyński, Piotr Wojtal, Wolfgang Müller, Luca Bondioli, Alessia Nava, Mihály Gasparik

|  | Trace elements | Sr isotope composition |
| --- | --- | --- |
| Laser ablation | RESOlution M-50 | |
| Wavelength | 193 nm (ArF) | |
| Pulse length (ns) | 20 | |
| Fluence at sample (J/cm^2^) | 7 | |
| Repetition rate (Hz) | 15 | 20 |
| Spot diameter (µm) | 67 | 120 |
| Line scan speed (mm/min) | 2 | 0.5 |
| Mass spectrometer | ICP MS XSeriesII | MC ICP-MS Neptune |
| RF power (W) | 1400 | 1250 |
| Sample gas Ar flow (L/min) | 0.8 - 0.9 | 0.8 - 0.9 |
| Cool gas Ar flow (L/min) | 13 | 15 |
| Auxiliary gas Ar flow (L/min) | 0.8 - 0.9 | 0.8 - 0.9 |
| Nitrogen flow (mL/min) | 5 - 10 | 5 - 10 |
| He flow (L/min) | ca. 0.3 | ca. 0.3 |
| Background measurements | 120 | 90 |
| Washout time (s) | 350 |  |
